# Application of systems biology-based in silico tools to optimize treatment strategy identification in Still’s disease

**DOI:** 10.1186/s13075-021-02507-w

**Published:** 2021-04-23

**Authors:** Cristina Segú-Vergés, Mireia Coma, Christoph Kessel, Serge Smeets, Dirk Foell, Anna Aldea

**Affiliations:** 1 Anaxomics Biotech, Carrer Diputació 237, 1°, 1ª, 08007 Barcelona, Catalonia Spain; 2 Department of Pediatric Rheumatology & Immunology, University Children’s Hospital, Albert-Schweitzer-Campus 1, 48149 Münster, Germany; 3 Novartis, Haaksbergweg, 1101 BX, Amsterdam, The Netherlands; 4 Novartis, Gran Via de les Corts Catalanes, 764, 08013 Barcelona, Catalonia Spain

**Keywords:** Systems biology, Systemic juvenile idiopathic arthritis, Adult-onset Still’s disease, Treat-to-target, Artificial intelligence, Machine learning, Personalized medicine

## Abstract

**Background:**

Systemic juvenile idiopathic arthritis (sJIA) and adult-onset Still’s disease (AOSD) are manifestations of an autoinflammatory disorder with complex pathophysiology and significant morbidity, together also termed Still’s disease. The objective of the current study is to set in silico models based on systems biology and investigate the optimal treat-to-target strategy for Still’s disease as a proof-of-concept of the modeling approach.

**Methods:**

Molecular characteristics of Still’s disease and data on biological inhibitors of interleukin (IL)-1 (anakinra, canakinumab), IL-6 (tocilizumab, sarilumab), and glucocorticoids as well as conventional disease-modifying anti-rheumatic drugs (DMARDs, methotrexate) were used to construct *in silico* mechanisms of action (MoA) models by means of Therapeutic Performance Mapping System (TPMS) technology. TPMS combines artificial neuronal networks, sampling-based methods, and artificial intelligence. Model outcomes were validated with published expression data from sJIA patients.

**Results:**

Biologicals demonstrated more pathophysiology-directed efficiency than non-biological drugs. IL-1 blockade mainly acts on proteins implicated in the innate immune system, while IL-6 signaling blockade has a weaker effect on innate immunity and rather affects adaptive immune mechanisms. The MoA models showed that in the autoinflammatory/systemic phases of Still’s disease, in which the innate immunity plays a pivotal role, the IL-1β-neutralizing antibody canakinumab is more efficient than the IL-6 receptor-inhibiting antibody tocilizumab. MoA models reproduced 67% of the information obtained from expression data.

**Conclusions:**

Systems biology-based modeling supported the preferred use of biologics as an immunomodulatory treatment strategy for Still’s disease. Our results reinforce the role for IL-1 blockade on innate immunity regulation, which is critical in systemic autoinflammatory diseases. This further encourages early use on Still’s disease IL-1 blockade to prevent the development of disease or drug-related complications. Further analysis at the clinical level will validate the findings and help determining the timeframe of the window of opportunity for canakinumab treatment.

**Supplementary Information:**

The online version contains supplementary material available at 10.1186/s13075-021-02507-w.

## Background

Still’s disease encompasses rare inflammatory disorders ranging from systemic juvenile idiopathic arthritis (sJIA), which occurs in children, to adult-onset Still’s disease (AOSD) in adults [1, 2]. The overlapping clinical features suggest that sJIA and AOSD are manifestations of a phenotypic continuum [2, 3]. Autoinflammatory features such as quotidian fever, polyserositis, evanescent rash, and substantial systemic inflammation dominate the clinical presentation of Still’s disease at its onset [1]. Interleukin (IL)-1β has been shown to drive initial inflammation [4]. In addition, high levels of IL-6 and IL-18 as well as S100A8/A9 and S100A12 are detectable in serum [1, 5]. sJIA can be complicated during the systemic phase by macrophage activation syndrome, a severe hyperinflammatory condition that can result in multi-organ failure with high mortality [6].

While the pathophysiology of Still’s disease involves a complex immune dysregulation on a polygenetic background, specific factors that trigger the disease are currently unknown. Today, Still’s disease is discussed as a biphasic disease [7]. Although a systemic and autoinflammatory phenotype is typically seen at the beginning, in later phases, patients often present extremely aggressive joint manifestations with destructive (poly)arthritis. A shift in molecular mechanisms and clinical presentation towards severe autoimmune arthritis appears to occur [8, 9]. In cases where it is possible to interrupt hyperinflammation in the initial phase, the development of chronic destructive joint inflammation can be prevented in the further course of the disease. Indeed, it has been demonstrated that IL-1 blockade is particularly effective when initiated as first-line therapy during the systemic phase [10, 11]. Further studies suggest that patients may benefit from therapeutic IL-6 receptor (IL6R) blockade [12], but head-to-head comparison of drugs interfering with either IL-1 or IL-6 signaling is still lacking.

Over the past years, it has been established that effective therapies including cytokine blockers should be initiated early in the disease course to exploit the “window of opportunity” [7]; however, not even in this case patients respond in a uniform manner. Consequently, “treat-to-target” protocols instruct switching therapies if treatment targets are missed and no clinical improvement is observed. It is currently impossible to choose the best options for individualized therapies upfront, e.g., by identifying molecular signatures indicating specific pathways active in patients with different phenotypes or endotypes. According to the success of selected therapeutic strategy, patients can be thus classified as presenting chronic, polycyclic, or monocyclic courses of the disease. The clinical complexity of the disease, with its dual systemic/rheumatic symptomatology, makes it challenging for clinicians to set an optimal treatment approach, as well as for researchers to identify clear molecular signatures for the proposed phenotypes; however, some recent initiatives are starting to address this issue [8]. Thus, the most urgent unmet need in the context of Still’s disease today is to find novel ways to advance the understanding of the pathophysiology underlying its clinical presentation and the molecular mechanisms of action (MoAs) of available therapies [8, 13].

In recent years, the application of bioinformatics and systems biology to generate pathophysiology-feasible models has generated great interest in drug development and regulatory decision fields for its potential in identifying optimal therapeutic strategies [14]. Systems biology- and machine learning-based methods are increasingly becoming a reliable strategy to understand the molecular effects of a pathology or drug in complex clinical settings, and they are already being used in the context of juvenile idiopathic arthritis [15, 16].

We hypothesized that systems biology-based models of Still’s disease, constructed with artificial intelligence techniques, can be employed to answer the most challenging questions regarding disease understanding, patient diagnosis, and therapeutic target approaches, as reported for other diseases. In this study, we used previously described protocols [17] to model the MoA of Still’s disease treatments in the context of human physiology, using systems biology- and artificial intelligence-based techniques. These models were used to deepen the understanding of the MoA of different therapeutic strategies, such as biological drugs that target the IL-1 pathway (anakinra and canakinumab) or the IL-6 pathway (tocilizumab and sarilumab, the latter not indicated for Still’s disease yet but being investigated in a phase II clinical trial, NCT02776735) and non-biological drugs (corticosteroids, i.e., prednisone, and methotrexate). The results obtained from the evaluation of these models and presented in this study provide a proof-of-concept of their applicability as research tools in this pathology and help to move towards the optimal treatment strategy of Still’s disease.

## Methods

### TPMS technology: Still’s disease systems biology-based model creation

Therapeutic performance mapping system (TPMS) is based on artificial intelligence and pattern recognition techniques that integrate all available biological, pharmacological, and medical knowledge to create mathematical models that simulate human (patho)physiology in silico. The methodology employed has been previously described [17].

**Fig. 1:**
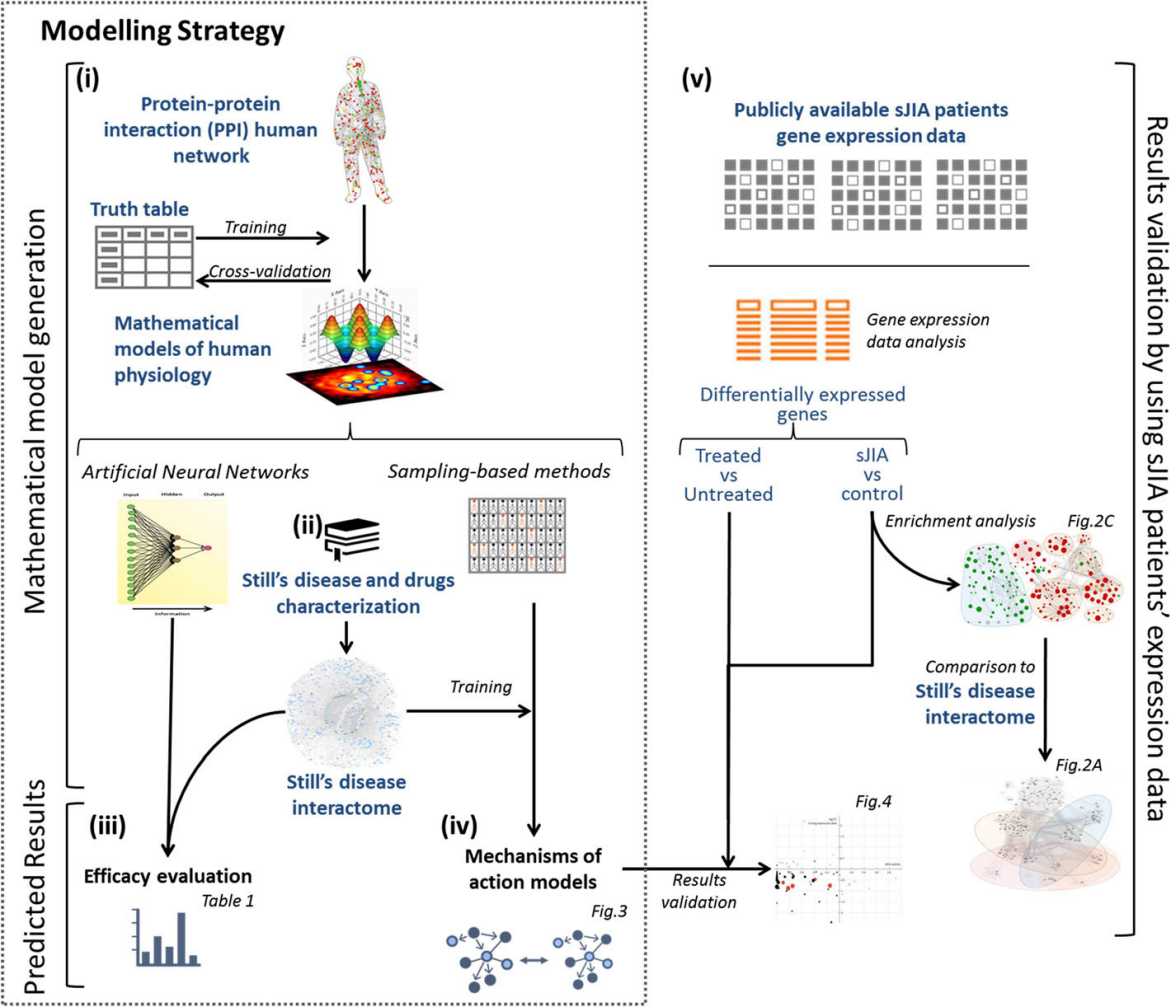
Schematic TPMS approach used to evaluate the Still’s disease treatments efficacy and their MoA. TPMS is based on systems biology-based models. TPMS encompass five steps: (i) the learning process of the protein-protein interaction (PPI) human network based on training and validation using known information stored in the truth table; this learning is performed with artificial intelligence techniques to construct accurate mathematical models that simulate the behavior of human physiology through two main strategies (ANNs and sampling-based methods). (ii) The molecular characterization of Still’s disease and drugs through dedicated bibliographical revision, from which a Still’s disease interactome can be constructed using the PPI human network. (iii) ANN evaluation of drugs efficacy over Still’s disease definition. (iv) The construction of specific MoA models for Still’s disease and drugs. (v) The validation of the MoA models with available gene expression data from sJIA patients. Expression data enrichment analysis was also used to be compared with the Still’s disease interactome to evaluate the validity of the bibliography-based Still’s disease molecular characterization

In this study, the models were focused on Still’s disease pathophysiology and the drug targets evaluated. Still’s disease pathophysiology was divided in known cellular and molecular processes, in order to help contextualize the model results according to the current disease knowledge. Still’s disease protein effectors and the drug protein targets were defined through manual curation of dedicated databases [18–21] and scientific literature (see supplementary methods, Additional file 1, and supplementary Tables S1-S3, Additional file 2). Models were trained using a compendium of biological and clinical data defining human physiology (supplementary Table S4, Additional file 2). Two modeling approaches were used: artificial neural networks (ANN), able to detect biological relationships, and sampling-based methods, able to explain biological relationships. Detailed information on the modeling methodology is provided in the supplementary methods, Additional file 1. TPMS-based models were used to evaluate the MoA and treatment efficacy in sJIA (Fig. 1).

### Expression data

All expression data from sJIA and AOSD patients available in Gene Expression Omnibus [22] until April 2018 were retrieved to be used for evaluating and biologically validating the models (see supplementary Table S5 [23–27], Additional file 2).

### Statistical analyses

Protein activity differences between the sampling-based MoA models were evaluated through Mann-Whitney-Wilcoxon test (multi-test correction using false discovery rate, significant results FDR < 0.05) and linear/quadratic classification analysis (significant results cross-validated accuracy > 80%).

Expression data from diseased individuals were evaluated using *t* test. Differentially expressed transcripts for sJIA evaluation were identified using T-Stouffer test for 4 experiments (T equivalent to FDR < 0.05). Differentially expressed transcripts for drug evaluation were identified using the paired samples *t* test (*P* value ≤ 0.05). Results from these analyses can be found in supplementary Tables S6-S8, Additional file 2.

Hypergeometric enrichment analysis was performed over the database-derived sJIA-related gene expression data (supplementary Table S6, Additional file 2) and the network around Still’s disease characterization to determine the presence of enriched pathways defined in functionally informative databases (see supplementary methods, Additional file 1). The degree of enrichment of the protein sets was evaluated for each database (FDR < 0.05).

### Validation of the results obtained from model analysis with expression data

The activity of the proteins presenting a differential (FDR < 0.05) behavior between canakinumab and tocilizumab was compared to sJIA-derived expression data. A validation score was calculated using the criteria detailed in the supplementary methods, Additional file 1, and a percentage over the total of potential validation points according to the validation design was calculated.

## Results

### Still’s disease interactome

**Fig. 2:**
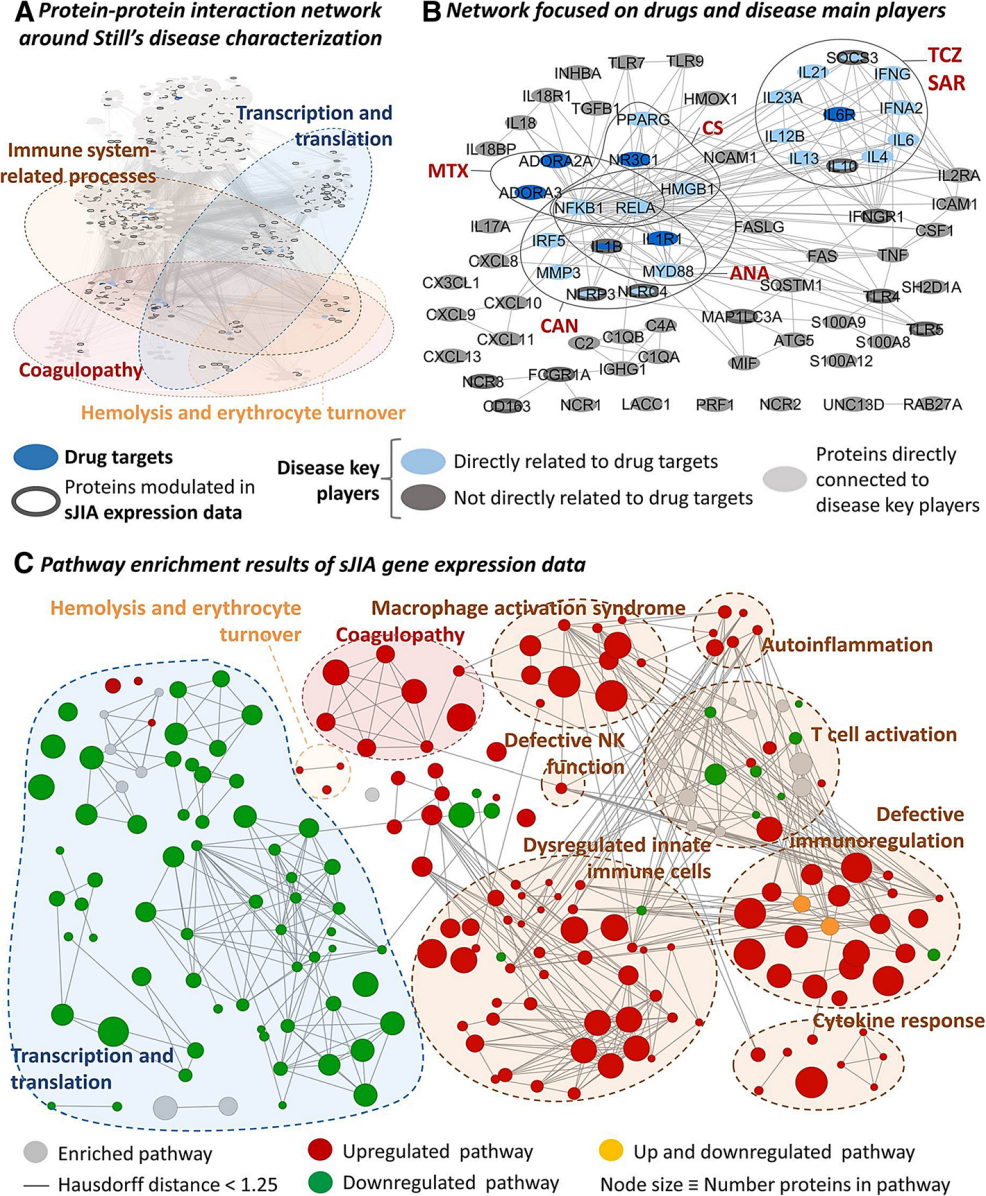
Still’ disease interactome **a** considering all disease effectors and their direct interactors and **b** focusing on disease effectors and their interaction to drug targets, and comparison to **c** pathway enrichment results of sJIA gene expression data. The network around Still’s disease characterization was constructed using the bibliographic characterization and interaction databases (**a**) presents enrichment of pathways found also enriched within the independent disease expression data (**c**). The network created around Still’s disease includes all drug targets evaluated in the study directly interacting with at least one effector of the disease (**b**). ANA, anakinra; CAN, canakinumab; CS, corticosteroids; MTX, methotrexate; SAR, sarilumab; TCZ, tocilizumab

The starting material for Still’s disease network generation was the list of proteins (effectors) obtained from literature review (supplementary Table S2, Additional file 2). The enrichment analysis of the proteins around Still’s disease effectors (Fig. 2a) reflected 72% of the processes that showed a significant enrichment when analyzing sJIA expression data sets (supplementary Table S6, Additional file 2). Immune-related processes and manifestations associated to the disease (hemolysis or coagulopathy) (Fig. 2c) are the most relevant for the study. Around half of the proteins included in Still’s disease network were associated to at least one of the commonly enriched processes. Furthermore, all treatments evaluated presented at least one target directly related to Still’s disease effectors (Fig. 2b).

### Efficacy evaluation of Still’s disease current treatment options

The ANN analysis showed a strong and significant relationship between the biological agents and the molecular pathophysiology of Still’s disease (> 78%, *p* < 0.05) and were quite similar to each other (Table 1). A weaker relationship was found between non-biological drugs and the molecular pathophysiology of Still’s disease. Similar results were obtained when the relationships between the different biological drugs and each Still’s disease component (systemic and rheumatic) was evaluated.

**Table 1:** Summary of ANN scores

		Biologics				Non-biologics	
		Anakinra	Canakinumab	Sarilumab	Tocilizumab	Methotrexate	Prednisone
**Still’s disease molecular definition**		**+++ (81%)**	**+++ (86%)**	**+++ (85%)**	**+++ (85%)**	− (5%)	++ (70%)
**Phenotypic profiles:**							
**Systemic profile**		**+++ (80%)**	**+++ (88%)**	**+++ (85%)**	**+++ (85%)**	− (4%)	++ (70%)
**Rheumatic profile**		**+++ (87%)**	**+++ (92%)**	**+++ (81%)**	**+++ (81%)**	− (9%)	++ (64%)
**Immune system components:**							
**Innate immune system deregulation**		++ (71%)	++ (71%)	+ (55%)	+ (55%)	− (10%)	++ (65%)
**Adaptive immune system**	**T cell response activation**	+ (45%)	− (37%)	++ (71%)	++ (71%)	− (25%)	+ (47%)
	**Defective immune regulation**	− (19%)	− (37%)	+ (47%)	+ (47%)	− (15%)	+ (50%)

ANN scores show the probability of the resulted relationship to be a true positive: +++ (highlighted in bold font) correspond to values > 78% (*p* value< 0.05); ++ correspond to values > 59% (*p* values < 0.15) and; + correspond to values > 38% (*p* value< 0.25). *ANN,* artificial neural network

In order to explore differences in the mechanisms of each drug, the individual motives implicated in Still’s disease pathophysiology (as defined by literature review, supplementary Table S1 and S2, Additional file 2) were also evaluated. ANN analyses showed that the action of the IL-1 blockers (anakinra and canakinumab) is related to the definition set for the innate immune system (supplementary tables S1-S2, Additional file 2), whereas the IL6R blockers (tocilizumab and sarilumab) are more related to the definition of the adaptive immune system (supplementary Tables S1-S2, Additional file 2) (Table 1).

As per the modeling approach used, tocilizumab and sarilumab are not distinguishable, as they share the pharmacological target. Both anakinra and canakinumab, acting over different points of the IL-1 pathway, also presented a similar behavior.

### Impact of IL-1- vs. IL-6-targeted treatment on proteins involved in the innate immune system response in Still’s disease

**Fig. 3:**
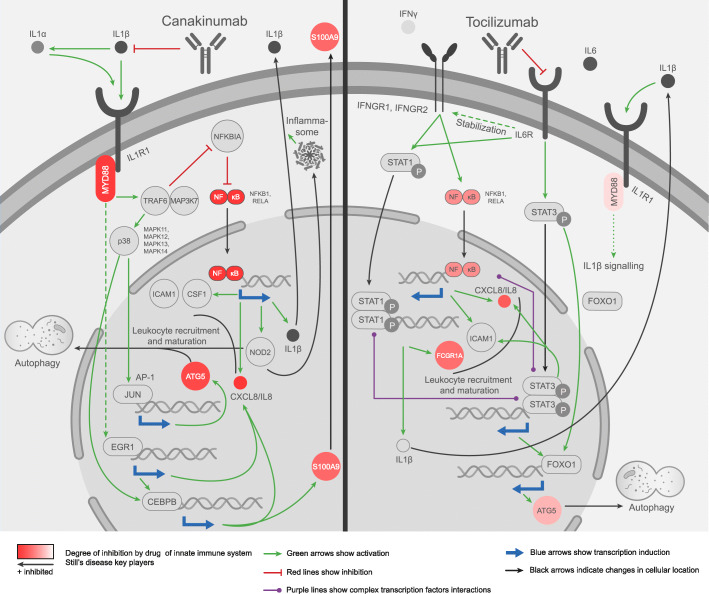
Systems biology-based MoA models of canakinumab and tocilizumab focused on innate immune system modulation. Canakinumab preferably modulates NF-κB, IL-8 (CXCL8), MyD88, S100A9, and ATG5, which are involved in processes of general innate immune inflammation, neutrophil recruitment, activation, and autophagy, whereas tocilizumab preferably modulates FCGR1, which is involved in neutrophil activation

Since Still’s disease onsets as an autoinflammatory disease, a deeper evaluation of the MoAs of canakinumab (as IL-1 inhibitor) and tocilizumab (as IL6R inhibitor) were performed focusing on the innate immune system effectors (list on supplementary Tables S1-S2, Additional file 2, and Table 2). The detailed evaluation of the MoA of canakinumab and tocilizumab on the innate immune system effectors showed that some well-known proteins were differentially modulated (Fig. 3). Most of them (13 out of total 16; 81.2%) were more strongly modulated to treat the disease by canakinumab than by tocilizumab (FDR < 0.001) (Table 2). Within these proteins, we selected those that were able to accurately classify the MoA solutions of each drug (cross-validated accuracy > 80%). The mechanisms of these “classifier” protein effectors were evaluated to explore their role within the mechanism of action of the drugs (Fig. 3). Supplementary Figure S1-S2, Additional file 3, and supplementary Tables S9-S10, Additional file 2, contain the sources of information found in the scientific literature supporting the predicted mechanisms. According to the models, canakinumab inhibits NF-κB (formed by the protein products of the genes NFKB1 and RELA), IL-8 (also known by its gene name, CXCL8), and S100A9 more effectively than tocilizumab, among other proteins. Canakinumab accomplishes this effect by preventing the interaction between IL-1β and its receptor IL-1R, with the subsequent inhibition of the canonical IL-1 signaling pathway. This pathway is initiated by the recruitment of the adaptor protein MyD88 to the IL-1R, which is known to involve the IRAK1-IRAK4-MyD88 complex formation and recruitment to the receptor and activation of E3 ubiquitin-protein ligase TRAF6 and mitogen-activated protein kinase MAP3K7, also known as TAK1. This signaling pathway transduces its signal through NF-κB and also through the p38 MAP kinase family (MAPK11-14) with the subsequent activation of AP-1 transcription factor. Aside from the activation of well-known pro-inflammatory mediators through the IL-1 signaling canonical pathway, the models show involvement of inhibition of the early growth response protein 1 (EGR1) and CCAAT enhancer binding protein beta (CEBPB) transcriptional activity in canakinumab mechanisms.

**Table 2:** Model activity values and differences between treatments among the innate immune system deregulation effector proteins

Gene name	Protein name	SD sign	Canakinumab	Tocilizumab	FDR	ACC
**FCGR1A**	High affinity immunoglobulin gamma Fc receptor I (CD64)	1	0	− 0.72	7.23E−38	0.995
**S100A9**	Protein S100-A9	1	− 0.75	0	2.03E−38	0.99
**NFKB1**	Nuclear factor NF-kappa-B p105 subunit	1	− 1	− 0.56	3.29E−36	0.98
**IL8**	Interleukin-8 (CXCL8)	1	− 1	− 0.82	9.50E−36	0.97
**MYD88**	Myeloid differentiation primary response protein MyD88	1	− 0.98	− 0.18	4.09E−33	0.955
**RELA**	Transcription factor p65 (TF65)	1	− 1	− 0.84	1.95E−33	0.925
**ATG5**	Autophagy protein 5	1	− 0.91	− 0.37	1.36E−26	0.865
**CSF1**	Macrophage colony-stimulating factor 1	1	− 0.89	− 0.73	7.23E−07	0.795
**SQSTM1**	Sequestosome-1	1	− 0.68	− 0.46	1.93E−12	0.795
**TLR7**	Toll-like receptor 7	-1	− 0.4	− 0.12	1.53E−07	0.745
**ICAM1**	Intercellular adhesion molecule 1	1	− 0.87	− 1	3.44E−14	0.73
**MIF**	Macrophage migration inhibitory factor	1	− 0.82	− 0.58	1.33E−04	0.72
**S100A12**	Protein S100-A12	1	0.16	− 0.13	1.63E−03	0.66
**S100A8**	Protein S100-A8	1	− 0.23	− 0.22	1.03E−01	0.635
**MAP1LC3A**	Microtubule-associated proteins 1A/1B light chain 3 (MLP3A)	1	− 0.28	− 0.04	1.80E−04	0.605
**INHBA**	Inhibin beta A chain	1	0.08	0	4.23E−03	0.57

SD sign column indicates whether the protein is increased/overactivated (1) or reduced/inhibited (− 1) in the context of Still’s disease. The columns canakinumab and tocilizumab present the predicted activity values (ranging from 1, totally activated, to − 1, totally inhibited) for each protein in each MoA model. FDR column indicates the false discovery rate obtained after Mann-Whitney-Wilcoxon test for each protein. The ACC column indicates the cross-validated accuracy of classification of the MoA models for each protein

In contrast, tocilizumab preferentially inhibits the Fc fragment of IgG receptor Ia or CD64 (protein codified by the gene known as FCGR1A). As shown in Fig. 3, tocilizumab prevents the interaction between the receptor subunit IL6R and its ligand IL-6, blocking the activation of the catalytic IL6R subunit gp-130. IL-6 signal is mediated through STATs, mainly through STAT3 activation; STAT3 is a signal transducer and transcription activator implicated in the expression of several IL-6 responsive genes. The models also show functional interactions between STAT3 and other transcription factors, such as FOXO1, which leads to modulation of autophagy protein 5 (ATG5), although with a lower potency than IL-1β blockade (Table 2 and Fig. 3). STAT3 and the NF-κB signaling pathways present a complex cross-talk, which is highlighted in the mechanism model for IL-6 blockade (Fig. 3 and Figure S2, Additional file 3); hence, we predicted a weaker inhibition of the NF-κB transcription activity (including, for example IL-8 (CXCL8)) by IL-6 blockade compared to IL-1β blockade. IL-6 blockade can also modulate STAT1 activity in two ways, through modulation of the stability of IFN-γ receptor mRNA (IFNGR1 and IFNGR2) and through regulatory cross-talk between STAT1 and STAT3. STAT1 modulation is responsible for FCGR1A transcription inhibition in the predicted tocilizumab mechanism of action.

### Validation of the drug-disease MoA models with expression data

**Fig. 4:**
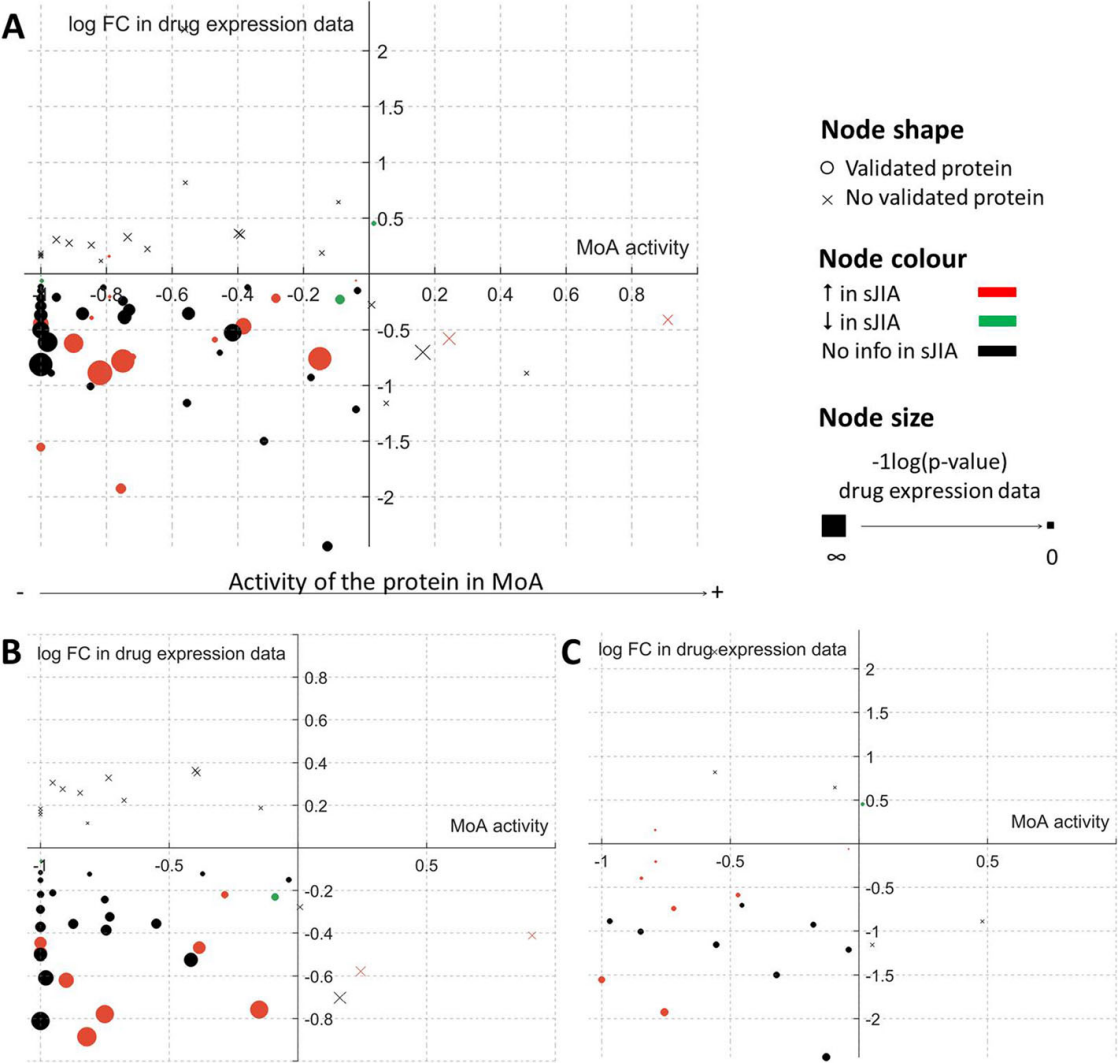
Validation of MoA models using expression data. Both sJIA vs. control (shown in nodes color) and treated vs. non-treated sJIA patients with canakinumab or tocilizumab expression data (*Y*-axis and node size) was used to compare model protein values (*X*-axis). Proteins validated by either sJIA vs. control or treated vs. non-treated expression data are represented by circles and non-validated proteins are represented by crosses. Most of the validated proteins fall in the third quadrant, i.e., their expression is downregulated by the drugs and the MoA model predict downregulation in their activation. The evaluation is represented considering **a** treated vs. non-treated sJIA patients with canakinumab or tocilizumab (each expression value matched with it corresponding MoA model value), **b** treated vs. non-treated sJIA patients with canakinumab (each expression value matched with canakinumab MoA model values), and **c** treated vs. non-treated sJIA patients with tocilizumab (each expression value matched with tocilizumab MoA model values)

Besides internal cross-validation of the models (94% accuracy), MoA models were validated using the sJIA gene expression data (analyzing both untreated- and treated- patients with canakinumab and tocilizumab). Figure 4 shows protein activities that are differential between canakinumab and tocilizumab MoA models and whether the predicted activity is validated or not by the change in expression induced by the treatment in sJIA patients (for both canakinumab and tocilizumab). Most validated model protein activities were predicted to be inhibited by the drugs and their expression was downregulated by the drugs. Most of these proteins presented gene expression upregulation in sJIA patients compared to controls. Overall, protein activities presenting significant differences between canakinumab and tocilizumab models (FDR < 0.05) reproduced a 67% of the information obtained from expression data.

## Discussion

This study shows how the application of systems biology-based *in silico* tools can help to identify improved treatment strategies of Still’s disease through providing a better understanding of their molecular mechanisms.

Currently, the therapy of Still’s disease is based on biological and non-biological therapeutic strategies. The in silico models reproduced known clinical and molecular findings of Still’s disease treatment. These models confirmed that the biological drugs tested as proof-of-concept had a more pathophysiology-directed MoA than non-biological drugs. This may reinforce the claims for a timely use of therapeutic approaches within treat-to-target protocols that include interleukin-blockade as first-line treatment, not only based on short-term responses but also because it supposes a reduction of long-term disease-associated complications, corticosteroid dependence, and potential chronic arthritic courses [7, 10, 12, 28–30]. Most importantly, the models provided feasible explanations of why IL-1β blockade may be an optimal therapeutic strategy for early-stage Still’s disease to prevent further disease progression.

Intriguingly, the mechanistic analysis suggests that both biological drug strategies (IL-1 and IL6R blockade) would be equally related to general Still’s disease pathophysiology, as well as the systemic and the rheumatic component. The scientific literature contains diverging evidence about the efficacy of each strategy (IL-1 and IL6R blockade) with respect to the different components of the disease (systemic/autoinflammatory and rheumatic/autoimmune). Some authors conclude that both strategies would be efficient in both settings [1, 31]. However, other studies provide evidence pointing towards the fact that IL-6 blockade would be more useful for the arthritis component of the disease [12, 31, 32], and still others suggest that the efficacy of IL-1 blockade would be limited to the systemic features of the disease [33–35]. The conflicting literature reports may be due to any of the following reasons: (i) the molecular mediators of both clinical components are closely related [36]; (ii) the lack of understanding of the mechanisms mediating each component; (iii) the true heterogeneity of the disease itself [1]; or (iv) biased information in the scientific literature about the pathophysiology, mainly focused on the arthritic phenotype and thus over-emphasizing this component. The acquisition and evaluation of more data on early Still’s disease patients’, as was recently performed by Gohar et al. [8], may advance understanding of the systemic component of the disease.

The predictive and mechanistic analyses suggest that biological drugs are effective by a combined action on different motives. The IL-1 blockers modulate the deregulation of innate immunity and well-known inflammatory proteins specifically. In contrast, IL6R blockers would rather act on t establishing the innate immune system deregulation loop, related to adaptive immunity, processes currently thought to happen in a later phase [7]. According to our models and the results observed in the evaluated proteins, IL-1 blockade might be more effective in treating deregulation of the innate immune system than IL6R blockers.

Due to the autoinflammatory nature of Still’s disease [9], deeper analysis on how IL-1 vs. IL6R blockers may affect the innate immune system was performed by modeling their mechanisms of action in detail, which were later biologically validated with expression data. Canakinumab was chosen as representative of IL-1 blockers because both, anakinra and canakinumab, showed similar results on the ANN analyses and canakinumab targets specifically the Still’s disease hallmark molecule IL-1β [4, 9], without affecting IL-1α. Similarly, tocilizumab was used as IL6R inhibitor representative because it is currently approved for sJIA treatment and because the expression data of sJIA patients required for the validation of the model are not available for sarilumab. According to the information used and the modeling approach, the conclusions from tocilizumab might be extended to sarilumab. The comparison analysis between IL-1β and IL6R inhibition showed that some well-known effector proteins were modulated at a different level by each strategy. Through the use of sampling methods-based models, canakinumab was predicted to inhibit more strongly most of the proteins included in the definition of innate response deregulation in Still’s disease than tocilizumab.

Canakinumab preferably modulates NF-κB, IL-8 (CXCL8), MyD88, S100A9, and ATG5 predicted activity; these proteins are involved in processes of general innate immune inflammation, neutrophil recruitment and activation, and autophagy, all of them processes involved in Still’s disease pathophysiology [9, 11, 37], These signaling is predicted to involve both canonical and non-canonical IL-1 downstream molecular pathways. In contrast, tocilizumab, through the collateral modulation of STAT1, preferably modulates FCGR1 activity, which is involved in neutrophil activation, a relevant hallmark in Still’s disease [11]. Furthermore, the IL-6 canonical downstream signaling partner, STAT3, has been extensively reported to interact with high complexity with NF-κB function in immune cells [38], and our models highlight this interaction as a relevant consequence of IL-6 blockade. These data are in line with the hypothesis that IL-1 blockade inhibits innate immune mechanisms more directly than IL-6 blockade. Using this ability to control disease at early stages, when innate immunity plays a key role [1, 4], might be a good option to prevent later disease- or treatment-derived complications.

Focused on Still’s disease, our study confirms the ability of systems biology *in silico* modeling approaches to provide mechanistic insights on the effects of current available drugs and grants their use to predict biomarkers of treatment response, required for tailoring treatment for the appropriate patients. Throughout the compilation and reinterpretation of available biological data, this *in silico* approach reproduces known aspects of the disease and the drugs and is able to generate new hypotheses. The presented technology relies on general human pathophysiological information, not only centered on the drugs and diseases under study. This aspect is of vital importance for rare diseases where patient numbers are low and detailed information on molecular pathophysiology is scarce, since it allows performing strategic studies including the available data without the need of large groups of patients. Similar studies performed in other clinical conditions demonstrate the potential clinical-translational use of TPMS [39, 40].

Nevertheless, the in silico modeling approaches and their biological validation are limited by the information about diseases, drugs, and the data available in public repositories. In the case of Still’s disease characterization, biased information in the scientific literature towards molecular definition of late or mixed (systemic and rheumatic) [8] might be shifting the results towards conclusions that are more based on arthritic/autoimmune versus systemic/autoinflammatory features. Likewise, the motive-based classification of the disease effectors in cellular and molecular processes is a simplification of the complexity that represents the immune system and its deregulation in the pathology. Regarding drug-related information, the expression data available was no comparable between the drugs due to different experimental designs (treatment time, number of samples, response criteria) and could not be used in the drugs’ MoA model construction. Given the modeling methodology applied, the fact of not including specific information on the different drugs allows for an unbiased evaluation of all the drugs tested. However, we used these data and other obtained from independent gene expression datasets, including a total of 197 sJIA patients and 79 control patients available in public repositories (supplementary Table S5, Additional file 2) to validate the models’ results. This validation has two main limitations: it compares different types of data (i.e., changes in gene expression data vs predicted protein activity) and there was lack of statistical power of expression data from tocilizumab-treated patients due to low number of samples. Furthermore, only sJIA (not AOSD) expression data was available and thus used for validation; however, as suggested by clinical [3, 33, 41, 42], epidemiological,^1^ and molecular data [2, 33, 41, 44, 49], we considered both pathological entities as a continuum, so sJIA data can be used to validate the Still’s disease models evaluation conclusions. Therefore, the models and conclusions are susceptible of being updated over time if prospective data and new information is generated (e.g., early sJIA patients’ datasets), making them more accurate regarding Still’s disease pathophysiology. With the current knowledge, we cannot define the time period after disease onset for the optimal efficacy of IL-1β blockade. The increasing trend of depositing data in public repositories to be shared among the scientific community will surely help performing more studies and accelerating clinical investigation [50], including the confirmation of the herein proposed hypotheses and the determination of the exact timeframe for canakinumab’s window of opportunity.

## Conclusions

In conclusion, the application of systems biology-based in silico modeling confirmed the use of biologics as an appropriate immunomodulatory treatment strategy for Still’s disease and supported the benefits of early IL-1 blockade to prevent the development of disease- or drug-related complications. Our data point towards a more efficient role of canakinumab in the initial autoinflammatory/systemic phases that are dominated by innate immune deregulation, which suggests that early interventions might allow to prevent the development of destructive (poly-)arthritis and long-term treatment-associated complications, although the exact timeframe of the window of opportunity for these interventions remains to be determined. Furthermore, the models propose detailed molecular mechanisms explaining the findings. All these results are in line with current knowledge and hypotheses on the mechanisms of Still’s disease treatment and represent a proof-of-concept of the applicability of systems biology-based artificial intelligence models in this clinical field.

## Supplementary Information

**Additional file 1: Supplementary methods.** Detailed explanation of the modeling methods applied.

**Additional file 2: Supplementary tables.** Includes from Supplementary Table S1 to S10. Contains information on scientific literature-based disease and drugs characterization, summary of information included in the truth table, list of publicly available gene expression datasets used for results contextualization and validation, results from the analysis of these datasets and bibliographic information supporting the interactions within the predicted mechanisms of action.

**Additional file 3: Supplementary figures.** Canakinumab and tocilizumab mechanism of action representation with supporting bibliographic references.

## Data Availability

All gene expression datasets analyzed during the current study are available in the Gene Expression Omnibus repository [GEO accession numbers: GSE80060, GSE17590, GSE21521, GSE8361 and GSE76492] [23–27], and the results of these analyses are included in this published article [and its supplementary information files]. All data compiled from the scientific literature to define the disease and drug under study and used in this study are included in this published article [and its supplementary information files].

## Abbreviations

ANN: Artificial neural networks
AOSD: Adult-onset Still’s disease
IL: Interleukin
IL6R: Interleukin 6 receptor
MoA: Mechanism of action
sJIA: Systemic juvenile idiopathic arthritis
TPMS: Therapeutic Performance Mapping System

## Footnotes

1 From the incidence point of view, both diseases present differences when age of onset [33, 43] or sex ratios [44–46] are taken into consideration. Differences in age are easily justified if we consider the infectious hypothesis [47]. In the case of sex, the hormonal influence justifies the fact that women are more likely to be affected by AOSD than men (70 vs. 30% in rheumatologic cohorts) [48]. Since children do not have sexual hormonal differences, this could explain the similar occurrence in boys and girls in sJIA.
