# Supplementary material for: Application of systems biology-based in silico tools to optimize treatment strategy identification in Still’s disease
Source: Arthritis Res Ther. 2021 Apr 23;23:126. doi: 10.1186/s13075-021-02507-w (PMC8063416; doi:10.1186/s13075-021-02507-w)
Supplement: Supplementary file 1 — Additional file 1: Supplementary methods. Detailed explanation of the modeling methods applied. [file 13075_2021_2507_MOESM1_ESM.pdf]

SUPPLEMENTARY METHODS

Application of Systems Biology-Based *In Silico* Tools to Optimize Treatment Strategy Identification in Still's Disease

AUTHORS: Cristina Segú-Vergés[a], Mireia Coma[a], Christoph Kessel[b], Serge Smeets[c], Dirk Foell[b], Anna Aldea[d]

AFFILIATIONS: [a]Anaxomics Biotech, Barcelona, Catalonia, Spain [b]Department of Pediatric Rheumatology & Immunology, University Children's Hospital, Münster, Germany [c]Novartis, Amsterdam, Netherlands [d]Novartis, Barcelona, Catalonia, Spain

Supplementary methods

TPMS technology: Systems biology-based model creation and analysis for Still's disease

A systems biology-based model was created and analysed by using Therapeutic Performance Mapping System (TPMS)[1] to investigate the Molecular Mechanisms of Action (MoA) of biological and non-biological drugs implicated in the modulation of Still's disease. TPMS is a validated top-down systems biology approach that integrates all available biological, pharmacological, and medical knowledge by means of pattern recognition and artificial intelligence techniques to create mathematical models that simulate *in silico* the behaviour of human physiology.

Molecular and biological characterization of Still's disease pathophysiology and drugs

For Still's disease characterization, we initiated an extensive and careful full-length review of relevant articles in the PubMed database (all until March, 2018) that consisted of the following search string: *((Still's disease) OR (systemic juvenile idiopathic arthritis) OR (juvenile idiopathic arthritis)) AND (molecular OR protein) AND (pathophysiology)*. The search was expanded using relevant references listed in the reviewed articles. The pathophysiological processes (motives) described to be involved in Still's disease (either as triggering, worsening or establishment facilitator factors) were identified, considering the most widely accepted biologically general concepts reported by the reviewed authors (supplementary Table S1). The motives were classified as participating in systemic or rheumatic phenotypic components of the disease, according to current knowledge. Subsequently, each motive was further functionally characterized at protein level to determine molecular effectors. A total of 65 unique proteins were identified to be functionally related to Still's disease according to scientific literature (supplementary Table S2). This process has been previously successfully applied to create models that have yielded experimentally validated conclusions[1].

For the drug molecular definition (anakinra, canakinumab, tocilizumab, sarilumab, prednisone, and metrotrexate), a revision of dedicated databases (DrugBank,[2, 3] STITCH,[4] SuperTarget[5]) and of scientific literature was performed (online supplementary Table S3).

Creation of human biological networks

The protein-protein interaction (PPI) human network created incorporated the available relationships (edges or links) between proteins (nodes) from a regularly updated in-house database drawn from public sources.[1] All information of the key proteins defined during the molecular and biological characterization, and stored in relevant databases (drug targets, other diseases effectors, biomarkers…) was incorporated into the biological networks.

Mathematical models generation

Biological maps were transformed into a mathematical model capable of both, reproducing existing knowledge and predicting new data. As it was deeply described,[1] and applied elsewhere [6-8], TPMS

---

[1] Giménez et al. (2020). Sci Rep DOI: 10.1038/s41598-020-78315-0
Lorén et al. (2019). J Crohns Colitis DOI: 10.1093/ecco-jcc/jjy171
Iborra-Egea et al. (2019). JACC Basic Transl Sci DOI: 10.1016/j.jacbts.2019.07.010
Romeo-Guitart et al. (2018).Sci Rep DOI: 10.1038/s41598-018-19767-3

technology uses a set of artificial intelligence algorithms to generate the human physiology over the human biological network [9-12].

Briefly, a selected collection of known input-output physiological signals considered the “truths” were collated into a table (truth table) to train de models [13]. The truth table was constructed using a compendium of biological and clinical databases [1-3, 14] through text mining techniques and manual review and curation of the information to obtain biological and pharmacological input-output relationships (such as drug-indication pairs). The biological or pathological conditions included in the truth table are molecularly characterized through specific scientific literature search and hand-curated assignment of proteins to the conditions (see *Molecular and biological characterization of Still’s disease pathophysiology and drugs* for information on Still’s disease characterization process). This information relating biological processes (adverse drug reactions, indications, diseases, and molecular pathways) to their molecular effectors, i.e. each one of the proteins involved in the physiological process, was compiled in the biological effectors database (BED) [1, 15]. The models had to be able to reproduce every rule contained in the truth table, and the error of a model is calculated as the sum of all the rules with which the model does not comply and the accuracy as the sum of all the rules complied with.

Thus, the approach allows creating models that integrate all the available biological, pharmacological, and medical knowledge, and are able to suggest mechanistic hypotheses that are consistent with actual biological processes.

Two complementary modelling strategies were used, Artificial Neural Networks (ANNs) and Sampling-based Methods, to compare the efficacy of the drugs and to elucidate the molecular mechanism of action (MoA).

ANNs are supervised algorithms that identify relations between proteins (e.g. drug targets) and clinical elements of the network [8, 11, 16] by inferring the probability of the existence of a specific relationship between two or more protein sets, based on a validation of the predictive capacity of the model towards the truth table. The learning methodology used consisted in an architecture of stratified ensembles of neural networks as a model, trained with a gradient descent algorithm to approximate the values of the given truth table. The neural network model used was a multilayer perceptron (MLP) neural network classifier. MLP gradient descent training depends on randomization initialization and to avoid random errors 1000 MLPs are trained with the training subset and the best 100 MLPs are used. In order to correctly predict the effect of a drug independently of the number of targets, different ensemble of neural networks are trained for different subset of drugs according to their number of targets (drugs with 1 target, 2 targets, 3 targets…). Then, the predictions for a query drug are calculated by all the ensembles, and pondered according to the number of targets of the query drug (the difference between the number of targets of the query and the number of targets of the drugs used to calculate each ensemble is used to ponder the result of each ensemble). A cross-validation with the truth table information showed that the accuracy of the described ANNs to reproduce the indications compiled in the truth table[1]) is 81.23% for

those drugs with all targets in the human biological network. This strategy was used for the efficacy evaluation of the biologic and non-biologic drugs respect Still's disease.

TPMS sampling-based methods generate models similar to a Multilayer Perceptron of an Artificial Neural Network over the human protein network (where neurons are the proteins and the edges of the network are used to transfer the information). This methodology was used for describing with high capability all plausible relationships between an input (or stimulus) and an output (or response). Sampling-based methods use optimization algorithms [10] to solve each parameter of the equation, i.e. the weights associated to the links between the nodes in the human protein network. In this approach, the network is limited by considering only interactions that connect drug targets with protein effectors in a maximum of three steps. The values of activation (+1) and inactivation (-1) of the targets of the drugs in the truth table were considered as input signals. The output results are the values of activation and inactivation of the proteins defining the phenotype (as retrieved from the BED). Each node of the protein network receives as input the output of the connected nodes in the direction flow from targets to effectors, weighted by each link weight. The sum of inputs is transformed by a hyperbolic tangent function to generate the score of the node (neuron), which become the "output signal" of the current node towards the nodes. The weight parameters are obtained by Stochastic Optimization Method based on Simulated Annealing, [10] which use probabilistic measures derived from the biological evidences to adjust network interaction types and strengths. Since the number of entries in the truth table is always smaller than the number of parameters (link weights) required by the algorithm, any process modelled by TPMS considers a population of different solutions. Models complying with the information in the truth table with a mean accuracy of 95.4% were obtained. In order to elucidate canakinumab/ tocilizumab vs. Still's disease innate immune system deregulation mechanisms of action, drug vs. disease-specific models were created by repeating the optimization process adding the new inputs (drug characterization) and output (disease characterization). The presented mechanisms of action are a mean representation of the solutions obtained, and it was checked whether each link was accurate, i.e., it was already described in the literature whether the MoA made sense overall, featuring pathways coherent with the living system and the known pathophysiology of Still's disease.

Expression data description and statistical analyses

Expression data from samples derived from sJIA patients (n= 197) and controls (n= 79), stored in Gene Expression Omnibus [17], were obtained and are summarised in supplementary Table S5) [18-22]. T tests were applied to determine differential expression and T-Stouffer test was employed for results unification when more than one experiment was considered for the same comparison [23].

Enrichment analysis

Hypergeometric enrichment analysis [24] was performed over the database-derived sJIA-related gene expression data (supplementary Table supplementary Table S6) and the network around Still's disease characterization to determine the presence of enriched pathways defined in functionally informative databases, including pathology-focused BED [1, 15]. pathway-oriented KEGG [25, 26], function-focused

Gene Ontology [27, 28], drug-related PHARMGKB[29] and SMPDB [30, 31], and transcription regulation-focused TRRUST[32] databases.

Validation of the results obtained from model analysis with expression data

The activity of the proteins presenting a differential (FDR<0.05) behaviour between canakinumab and tocilizumab was compared to sJIA vs. healthy patients and sJIA-treated patients expression data. A validation score was calculated using the following criteria, and a percentage over the total of potential validation points according to the validation design was calculated:

- genes with significant difference in the sJIA vs. healthy patients expression comparison (T-Stouffer equivalent to FDR<0.05) AND opposed modulation sign respect the drug *in silico* MoA where considered validated and assigned +1 validation point;
- genes with significant difference in the sJIA vs. healthy patients expression comparison (T-Stouffer equivalent to FDR<0.05) OR significant difference in the sJIA-treated analyses (p-value < 0.05) AND same modulation sign between drug-derived sJIA-treated patients expression analysis respect the drug *in silico* MoA predictions where considered validated and assigned +1 validation point;
- genes with tendency to difference in the sJIA-treated analyses (p-value < 0.3) AND same modulation sign between drug-derived sJIA-treated patients expression analysis respect the drug *in silico* MoA predictions where considered validated and assigned +0.5 validation points.

Supplementary references

1. Jorba, G., et al., *In-silico simulated prototype-patients using TPMS technology to study a potential adverse effect of sacubitril and valsartan.* PLoS One, 2020. 15(2): p. e0228926.
2. Wishart, D.S., et al., *DrugBank 5.0: a major update to the DrugBank database for 2018.* Nucleic Acids Res, 2018. 46(D1): p. D1074-D1082.
3. Wishart, D.S., et al., *DrugBank: a knowledgebase for drugs, drug actions and drug targets.* Nucleic Acids Res, 2008. 36(Database issue): p. D901-6.
4. Szklarczyk, D., et al., *STITCH 5: augmenting protein-chemical interaction networks with tissue and affinity data.* Nucleic Acids Res, 2016. 44(D1): p. D380-4.
5. Hecker, N., et al., *SuperTarget goes quantitative: update on drug-target interactions.* Nucleic Acids Res, 2012. 40(Database issue): p. D1113-7.
6. Loren, V., et al., *ANP32E, a Protein Involved in Steroid-Refractoriness in Ulcerative Colitis, Identified by a Systems Biology Approach.* J Crohns Colitis, 2019. 13(3): p. 351-361.
7. Iborra-Egea, O., et al., *Mechanisms of action of sacubitril/valsartan on cardiac remodeling: a systems biology approach.* NPJ Syst Biol Appl, 2017. 3: p. 12.
8. Romeo-Guitart, D., et al., *Neuroprotective Drug for Nerve Trauma Revealed Using Artificial Intelligence.* Sci Rep, 2018. 8(1): p. 1879.

9. Kirkpatrick, S., C.D. Gelatt, Jr., and M.P. Vecchi, *Optimization by simulated annealing.* Science, 1983. **220**(4598): p. 671-80.
10. Collet, P. and J.-P. Rennard, *Stochastic Optimization Algorithms.* ArXiv. abs/0704.3780.
11. Bishop, C.M., *Pattern Recognition and Machine Learning (Information Science and Statistics).*
12. Russell, S. and P. Norvig, *Artificial Intelligence: A Modern Approach*. 2009: Prentice Hall Press.
13. Anellis, I.H., *Peirce's Truth-functional Analysis and the Origin of the Truth Table.* History and Philosophy of Logic, 2012. **33**(1): p. 87-97.
14. Kuhn, M., et al., *The SIDER database of drugs and side effects.* Nucleic Acids Res, 2016. **44**(D1): p. D1075-9.
15. *Biological Effectors Database. TPMS technology, Anaxomics Biotech SL.*; Available from: http://www.anaxomics.com/biological-effectors-database.php.
16. Pujol, A., et al., *Unveiling the role of network and systems biology in drug discovery.* Trends Pharmacol Sci, 2010. **31**(3): p. 115-23.
17. Barrett, T., et al., *NCBI GEO: archive for functional genomics data sets--update.* Nucleic Acids Res, 2013. **41**(Database issue): p. D991-5.
18. Hinze, C.H., et al., *Immature cell populations and an erythropoiesis gene-expression signature in systemic juvenile idiopathic arthritis: implications for pathogenesis.* Arthritis Res Ther, 2010. **12**(3): p. R123.
19. Brachat, A.H., et al., *Early changes in gene expression and inflammatory proteins in systemic juvenile idiopathic arthritis patients on canakinumab therapy.* Arthritis Res Ther, 2017. **19**(1): p. 13.
20. Quartier, P., et al., *A multicentre, randomised, double-blind, placebo-controlled trial with the interleukin-1 receptor antagonist anakinra in patients with systemic-onset juvenile idiopathic arthritis (ANAJIS trial).* Ann Rheum Dis, 2011. **70**(5): p. 747-54.
21. Ishikawa, S., et al., *Abnormal expression of the genes involved in cytokine networks and mitochondrial function in systemic juvenile idiopathic arthritis identified by DNA microarray analysis.* Ann Rheum Dis, 2009. **68**(2): p. 264-72.
22. Omoyinmi, E., et al., *Mitochondrial and oxidative stress genes are differentially expressed in neutrophils of sJIA patients treated with tocilizumab: a pilot microarray study.* Pediatr Rheumatol Online J, 2016. **14**(1): p. 7.
23. Stouffer, S.A., *A study of attitudes.* Sci Am, 1949. **180**(5): p. 11-5.
24. Rivals, I., et al., *Enrichment or depletion of a GO category within a class of genes: which test?* Bioinformatics, 2007. **23**(4): p. 401-7.
25. Kanehisa, M., et al., *KEGG: new perspectives on genomes, pathways, diseases and drugs.* Nucleic Acids Res, 2017. **45**(D1): p. D353-D361.
26. Kanehisa, M. and S. Goto, *KEGG: kyoto encyclopedia of genes and genomes.* Nucleic Acids Res, 2000. **28**(1): p. 27-30.

27. Ashburner, M., et al., *Gene ontology: tool for the unification of biology. The Gene Ontology Consortium.* Nat Genet, 2000. **25**(1): p. 25-9.
28. The Gene Ontology, C., *Expansion of the Gene Ontology knowledgebase and resources.* Nucleic Acids Res, 2017. **45**(D1): p. D331-D338.
29. Whirl-Carrillo, M., et al., *Pharmacogenomics knowledge for personalized medicine.* Clin Pharmacol Ther, 2012. **92**(4): p. 414-7.
30. Frolkis, A., et al., *SMPDB: The Small Molecule Pathway Database.* Nucleic Acids Res, 2010. **38**(Database issue): p. D480-7.
31. Jewison, T., et al., *SMPDB 2.0: big improvements to the Small Molecule Pathway Database.* Nucleic Acids Res, 2014. **42**(Database issue): p. D478-84.
32. Han, H., et al., *TRRUST: a reference database of human transcriptional regulatory interactions.* Sci Rep, 2015. **5**: p. 11432.
