# Supplementary material for: Application of systems biology-based in silico tools to optimize treatment strategy identification in Still’s disease
Source: Arthritis Res Ther. 2021 Apr 23;23:126. doi: 10.1186/s13075-021-02507-w (PMC8063416; doi:10.1186/s13075-021-02507-w)
Supplement: Supplementary file 3 — Additional file 3: Supplementary figures. Canakinumab and tocilizumab mechanism of action representation with supporting bibliographic references. [file 13075_2021_2507_MOESM3_ESM.pdf]

SUPPLEMENTARY FIGURES

Application of Systems Biology-Based *In Silico* Tools to Optimize Treatment Strategy Identification in Still's Disease

AUTHORS: Cristina Segú-Vergés[a], Mireia Coma[a], Christoph Kessel[b], Serge Smeets[c], Dirk Foell[b], Anna Aldea[d]

AFFILIATIONS: [a]Anaxomics Biotech, Barcelona, Catalonia, Spain [b]Department of Pediatric Rheumatology & Immunology, University Children's Hospital, Münster, Germany [c]Novartis, Amsterdam, Netherlands [d]Novartis, Barcelona, Catalonia, Spain

Supplementary figures

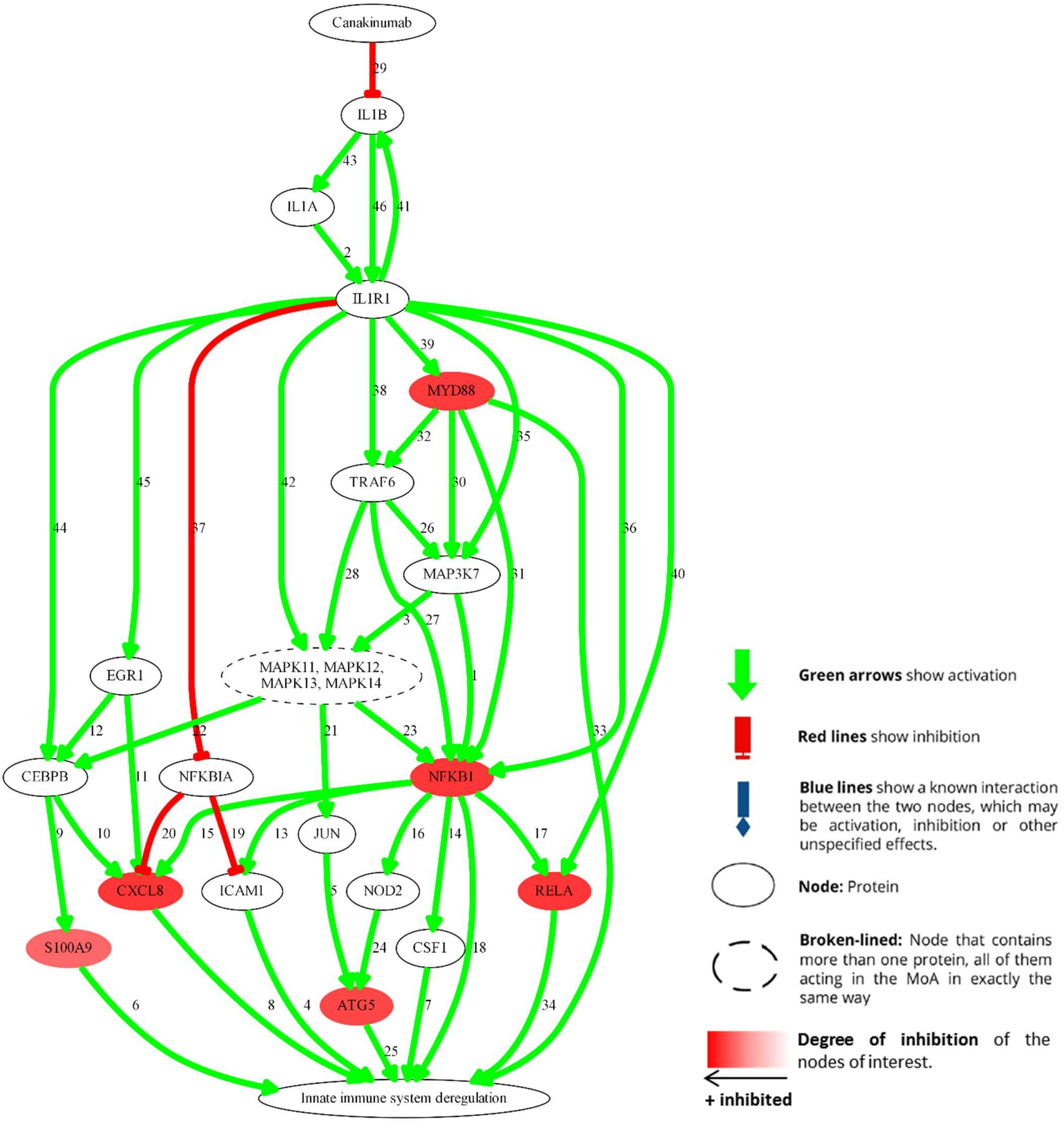

Supplementary Figure S1. Schematic representation of predicted canakinumab mechanism of action over Still's disease innate immune system deregulation. The number on each link indicates to the number of the corresponding entry on Table S9, to retrieve the sources of information found in the scientific literature supporting the predicted mechanisms. Figure created to represent TPMS mechanism of action predictions using Graphviz software (https://graphviz.gitlab.io/).

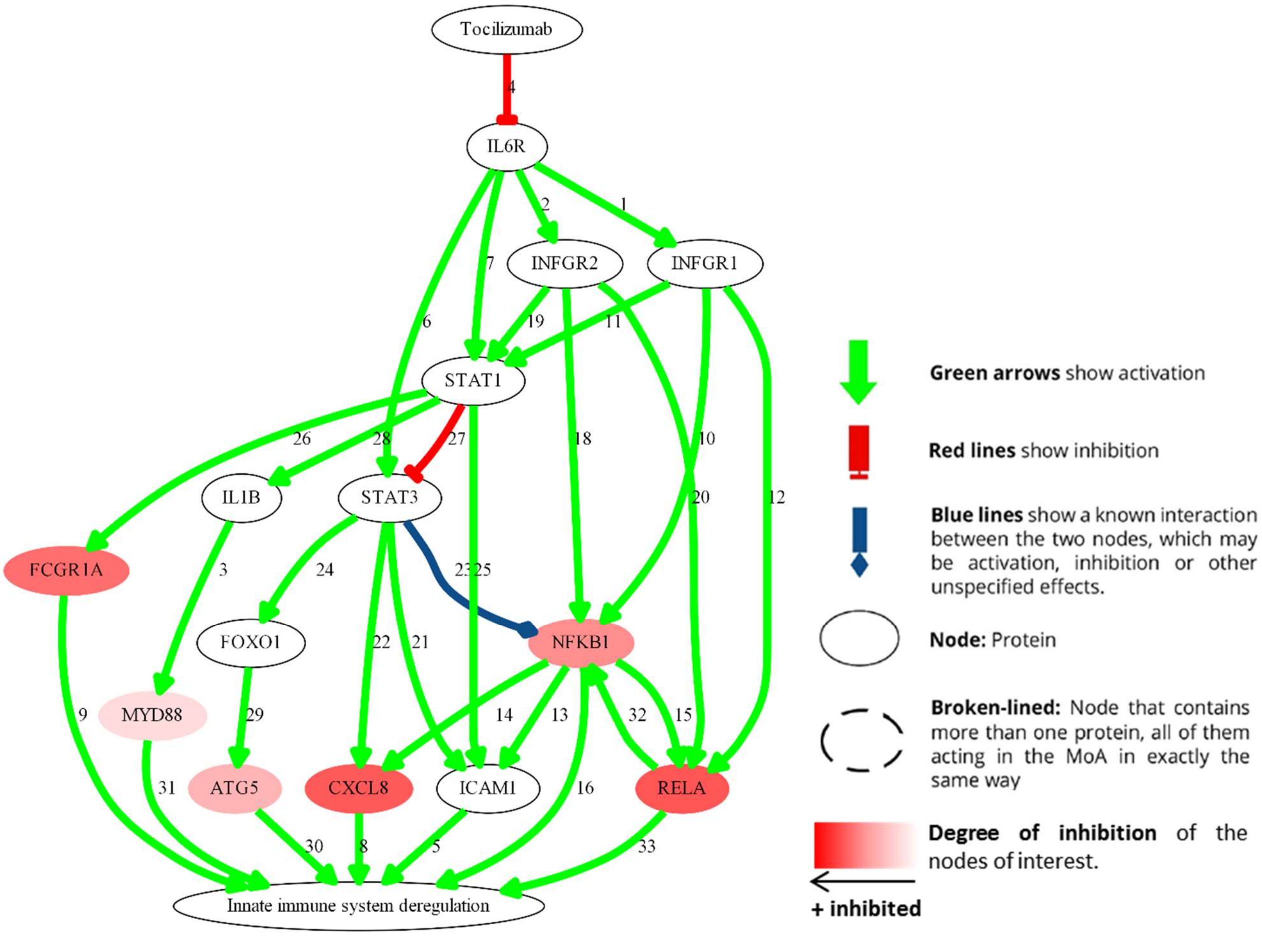

Supplementary Figure S2. Schematic representation of predicted tocilizumab mechanism of action over Still's disease innate immune system deregulation. The number on each link indicates to the number of the corresponding entry on Table S10, to retrieve the sources of information found in the scientific literature supporting the predicted mechanisms. Figure created to represent TPMS mechanism of action predictions using Graphviz software (https://graphviz.gitlab.io/).
